# Characterization of Polyvinylidene Fluoride (PVDF) Electrospun Fibers Doped by Carbon Flakes

**DOI:** 10.3390/polym12122766

**Published:** 2020-11-24

**Authors:** Pavel Kaspar, Dinara Sobola, Klára Částková, Alexandr Knápek, Daniel Burda, Farid Orudzhev, Rashid Dallaev, Pavel Tofel, Tomáš Trčka, Lubomír Grmela, Zdeněk Hadaš

**Affiliations:** 1 Department of Physics, Faculty of Electrical Engineering and Communication, Brno University of Technology, Technická 2848/8, 616 00 Brno, Czech Republic; kasparp@feec.vutbr.cz (P.K.); sobola@feec.vutbr.cz (D.S.); burda@isibrno.cz (D.B.); xdalla03@stud.feec.vutbr.cz (R.D.); tofel@feec.vutbr.cz (P.T.); trcka@feec.vutbr.cz (T.T.); grmela@vutbr.cz (L.G.); 2 Central European Institute of Technology BUT, Purkyňova 123, 612 00 Brno, Czech Republic; klara.castkova@ceitec.vutbr.cz; 3 Department of Inorganic Chemistry and Chemical Ecology, Dagestan State University, Makhachkala, St. M. Gadjieva 43-a, 367015 Dagestan Republic, Russia; farid-stkha@mail.ru; 4 Department of Ceramics and Polymers, Faculty of Mechanical Engineering, Brno University of Technology, Technická 2, 616 69 Brno, Czech Republic; 5 Institute of Scientific Instruments of the Czech Academy of Sciences, Královopolská 147, 612 64 Brno, Czech Republic; 6 Faculty of Mechanical Engineering, Brno University of Technology, Technická 2896/2, 616 69 Brno, Czech Republic; hadas@fme.vutbr.cz

**Keywords:** polyvinylidene fluoride, graphite, scanning electron microscopy, X-ray diffraction, Raman spectroscopy, Fourier-transform infrared spectroscopy, triboelectric effect

## Abstract

Polyvinylidene fluoride (PVDF) is a modern polymer material used in a wide variety of ways. Thanks to its excellent resistance to chemical or thermal degradation and low reactivity, it finds use in biology, chemistry, and electronics as well. By enriching the polymer with an easily accessible and cheap variant of graphite, it is possible to affect the ratio of crystalline phases. A correlation between the ratios of crystalline phases and different properties, like dielectric constant as well as piezo- and triboelectric properties, has been found, but the relationship between them is highly complex. These changes have been observed by a number of methods from structural, chemical and electrical points of view. Results of these methods have been documented to create a basis for further research and experimentation on the usability of this combined material in more complex structures and devices.

## 1. Introduction

Polymers are very topical materials useful in a great number of scientific fields. They are sought after for their excellent properties, namely high chemical stability and biocompatibility [1]. One of the more interesting materials that has gained a lot of popularity amongst scientists in the last couple of years, is polyvinylidene fluoride (PVDF). The great properties of this polymer, like low reactivity and high degree of thermoplasticity, as well as low cytotoxicity or chemical reactivity [2], have secured it a place in many areas, like semiconductors, biology, or chemistry. PVDF, though it can be difficult to manufacture and prepare, has a great potential for application in many areas of science and life. Preparation of PVDF requires a specialized approach, because preparation methods used for other polymers are not capable of producing homogeneous and usable products [3,4]. To create a PVDF fiber, methods like spin coating or electrospinning, are used to manufacture fibers with stable and repeatable results. Electrospinning specifically has been used in the case of this paper, as it allows for control over fiber diameter, inclusions, and crystalline phase to a certain extent [5].

Polyvinylidene fluoride has seen almost immediate use and a great deal of modification, further improving on its properties. By creating nanocomposites of the PVDF films with other materials, it is possible to increase their performance in difficult environments and to preserve and enhance their functional abilities as sensors and actuators [6,7]. Among the most popular materials for combination with PVDF is carbon. There are many papers focusing on the creation of hybrid composite films from PVDF and carbon [8], to use with carbon quantum dots for creation of films [9], or to use carbon with PVDF to improve its piezoelectric properties [10,11]. The focus of this paper is, however, not on films, but rather on PVDF fibers, and their combination with carbon. Research has been conducted into enhancing PVDF in both fiber and film form with carbon nanofibers already, [12,13], and even on carbonization of the PVDF fiber itself [14]. Graphene nanoparticles are among the inclusions for PVDF as well [15]. In this paper, we have decided to focus on carbon as well, but to use a much simpler and cheaper method in comparison to nanoparticles. Graphite flakes, a highly accessible and cheap material, are introduced into the fibers during their creation, which incorporates them into the fiber structure and changes their properties as well. To properly understand all the changes this incorporation of graphite flakes causes in PVDF, the pure original materials and their combination have been subjected to several measurements, namely scanning electron microscopy (SEM) to capture the topography, Raman spectroscopy, X-ray diffraction measurement (XRD), X-ray photoelectron spectroscopy (XPS), and Fourier-transform infrared spectroscopy (FTIR) to describe the properties and their changes on chemical level, and permittivity measurement to observe the electrical properties. The overarching reason for these experiments is that there are several benefits to changing the concentration of phases. α phase has higher dielectric constant and can therefore be used, where high energy storage properties are required. When combined with a relatively high breakdown voltage of the polymer, controlling the dielectric constant can be of great interest. β phase, on the other hand, lowers the dielectric constant, but introduces piezoelectric activity into the material and modifies triboelectric properties, which can then be used for energy harvesting or storage related purposes [16]. The ability to dictate the concentration of crystalline phases and dielectric and piezoelectric properties linked to them is very important for the maximalization of potential usefulness of the material. The novelty of this work is in the use of low-cost carbon materials and description of structure and properties in dependence on the powder concentration. We show that the incorporation of carbon powder as a filler into PVDF, even without functionalization and surface activation of the powder, allows for the control of properties of the resulting composite. This work contributes to the study of the formation of complex polymer systems and its modifications.

## 2. Materials and Methods

The main material in question is PVDF (Mw = 275.000 g/mol, Sigma Aldrich, Munich, Germany), more specifically, PVDF fibers. These fibers were manufactured by the method of electrospinning from 15 wt% PVDF solution in dimethylsulfoxide-aceton blend in volume ratio of 7/3 under a constant voltage of 50 kV, creating a fiber mat with the thickness of around 25 µm. The PVDF, or graphite doped PVDF solutions, were electrospun using 4-spin electrospinning equipment (Contipro, Dolní Dobrouč, Czech Republic) at a feeding rate of 20 µl.min^−1^ through a needle with an inner diameter of 1.067 mm (17 G) on a rotation collector covered by aluminum foil at a speed of 2000 rpm for 30 min. The distance between the needle tip and the collector was 20 cm. The fibers were collected in the form of non-woven mats, which were dried at laboratory temperature overnight. The whole process yielded resulting fibers with the width of 195.2 nm. In the case of enriched fibers, the 1% wt of graphite flakes were added into the polymer solution and the electrospinning process was performed with the same properties as with the pure PVDF solution.

Graphite powder is of the D50 grade with size of the particles of 3.5 µm. The powder was purchased from GK Graphite and the parameters were taken from their documentation. This material is widely accessible and is the same grade used for manufacture of pencil lead.

Raman spectroscopy was performed using WITec alpha300 R device (WITec, Ulm, Germany). Excitation wavelength for the experiments was set at 532 nm. Power of the laser used for excitation of the samples was 0.5 mW for the graphite flakes and PVDF fibers with the flakes, whereas for PVDF fibers without the flakes the power had to be increased to 1 mW to obtain a viable signal to noise ratio. The resulting signal was reconstructed from 50 accumulations with the integration time of 20 s.

SEM images were captured by a high-resolution scanning electron microscope FEI Verios 460 L (FEI, Brno, Czech Republic). To allow the use of SEM and improve the quality of obtained images, samples of pure PVDF and those with graphite flakes were covered with a 15 nm thick carbon film to improve their conductivity. The coating was done by carbon thread evaporator Leica EM ACE600. Carbon was used because of its accessibility and to prevent artefacts in the image due to conglomeration of golden particles during coating. Voltage of the electron beam was kept at 5 kV during the whole process, whereas the current was adjusted according to circumstances to receive the best images possible. In addition to the “through-lens detector” (TLD), charge neutralization mode (CN) had to be used as well for some samples, where charging of the edges was an issue.

XPS spectra were taken by AXIS Supra X-ray photoelectron spectrometer (Kratos Analytical, Manchester, UK). Emission current used to capture the resulting information was set at 15 mA. During the process, resolution 20 was utilized to for the measurement of wide spectra, and 80 for the element spectra. Final fitting of the spectra was performed using CasaXPS software.

FTIR measurement was taken in transmission mode (Bruker, Billerica, MA, USA), with resolution of 1 cm^−1^, over 512 iterations.

XRD analysis was done using X-ray powder diffractometer Rigaku SmartLab 3 kW (Rigaku Corporation, Tokyo, Japan) in Bragg-Brentano configuration was performed. Diffraction patterns were measured from 10° to 50° (2θ) with Cu Kα radiation.

Dielectric measurement was performed with Novocontrol Alpha Analyzer (Novocontrol Technologies, Montabaur, Germany) in the range between 1 Hz and 100,000 Hz in room temperature for the acquisition of basic information about dielectric properties, mainly permittivity of the material.

Triboelectric energy performance of the proposed materials was evaluated by electrometer 6517b (Keithley, Solon, Ohio, USA). The triboelectric generator was assembled in vertical contact-separation mode. Moving part consisting of Cu electrode was controlled by vibration test system TV 50,018 (Tira, Schalkau, Germany). Sample was clamped on a fixed Cu electrode. The area of the active part of the generator was 30 × 30 mm. Mechanical force was measured by sensor 208C01 (PCB Piezotronics, Huckelhoven, Germany) and this sensor was situated on the side of fixed electrode. Displacement between electrodes was measured via interferometer ILD 1402-10 (Micro Epsilon, Ortenburg, Germany).

All of the methods described above, except for temperature dependent ones, were taken in room temperature.

## 3. Results and Discussion

**Figure 1:**
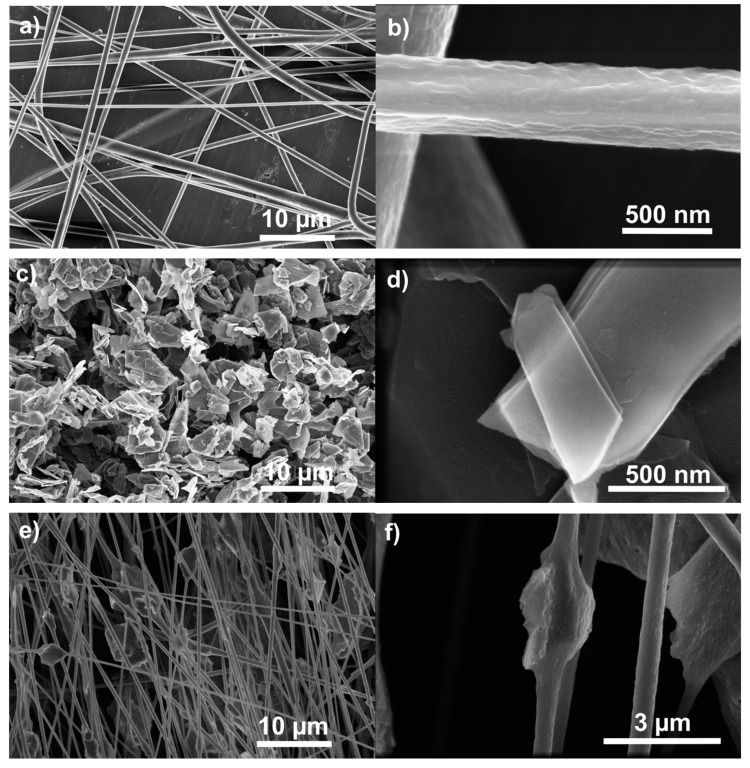
Pure PVDF Fibers, wide (**a**), and detail (**b**). Graphite flakes, wide (**c**) and detail (**d**) PVDF fibers with graphite flakes, wide (**e**) and detail (**f**).

To get a basic idea about the structural composition of the explored materials, SEM measurement was conducted on the three stages of the samples: pure PVDF, carbon flakes, and PVDF fibers with carbon flakes (Figure 1). Since the manufacturing process of PVDF fibers allows for the introduction of other materials into the source solution, the carbon flakes are incorporated into the structure of the fibers and thus become a part of it. Not only does this mean, that the flakes cannot be extracted from the composite by basic mechanical perturbations, like shaking or twisting of the fiber, but also that the structure of the polymer had to adapt and engulf the flakes. This can be best seen in detail in Figure 1b,d,f, depicting a focused detail of a single PVDF fiber, a carbon flake, and the resulting incorporated flake into the fiber, respectively.

**Figure 2:**
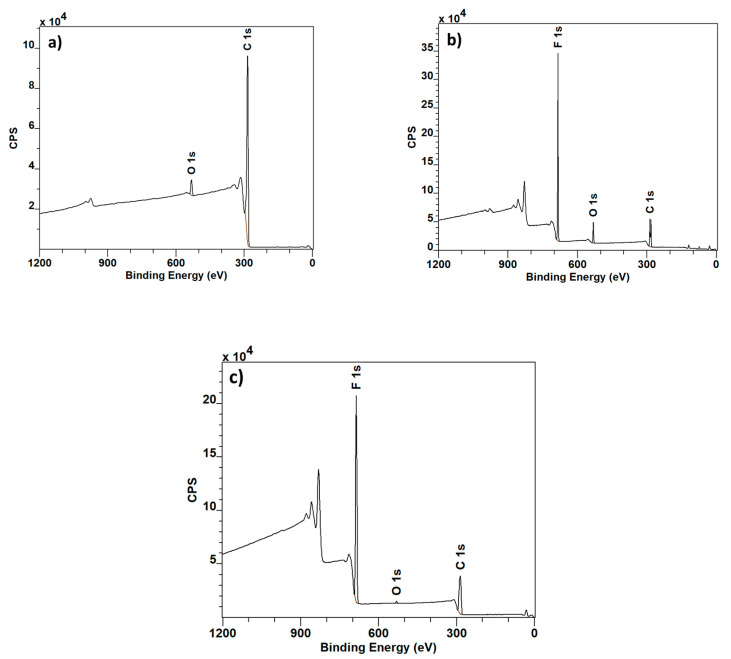
Wide XPS spectra: Graphite (**a**), PVDF (**b**) and PVDF with Graphite (**c**).

The samples were subjected to XPS measurement. Figure 2a–c show the wide spectra of pure carbon flakes, pure PVDF and PVDF combined with carbon flakes, respectively. In the combined material (Figure 2c) the proportional content of carbon is slightly higher, which is to be expected from the addition of carbon flakes. In the pure carbon spectrum (Figure 2a), it is possible to spot a peak belonging to oxygen, likely caused by oxidization of the surface or residual adsorbed moisture.

**Figure 3:**
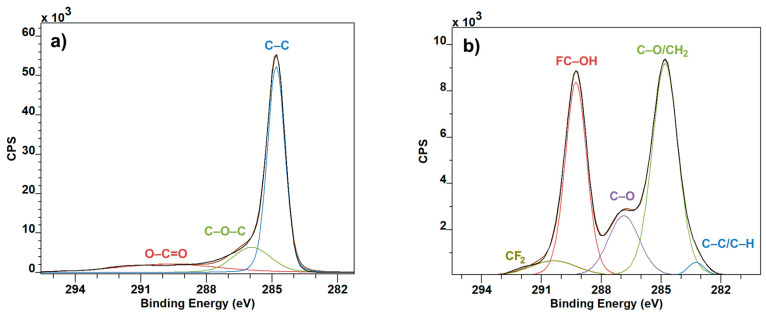

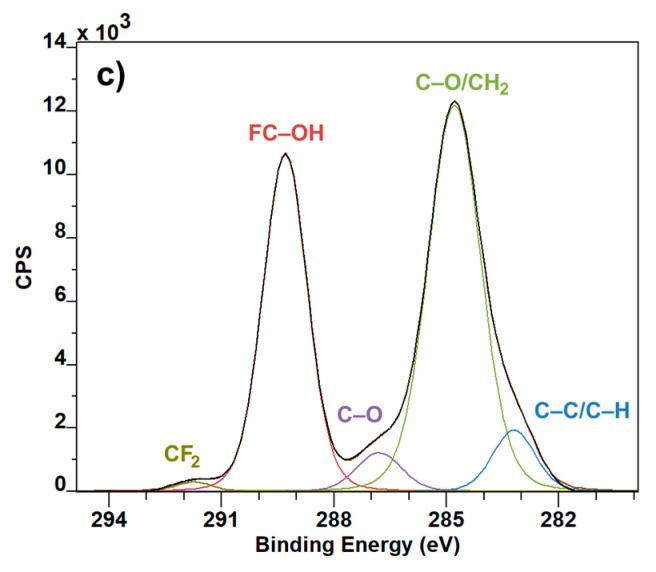
C1 XPS spectra: Graphite (**a**), PVDF (**b**) and PVDF with Graphite (**c**).

From the XPS measurement it can be seen that the XPS spectrum of pure graphite flakes (Figure 3a) is very much like it can be expected [17,18]. The C–C peak at 285 eV is a staple of graphite, for nano size or bigger as well. This presence can be seen altering the dominant C–O/CH_2_ peak of PVDF (Figure 3c), making it wider, and taking over from the C–O peak at 287 eV (Figure 3b). This is the most visible change in the XPS spectrum from the point of view of C1 peaks.

**Figure 4:**
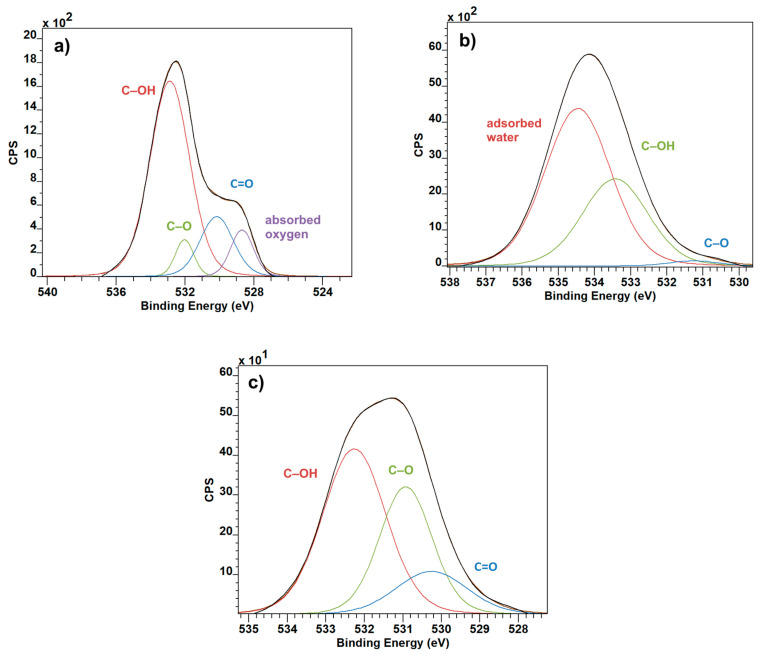
O1 XPS spectra: Graphite (**a**), PVDF (**b**) and PVDF with Graphite (**c**).

The greatest inconsistencies can be seen in the O1 spectra. The easily recognizable C=O bond at 529 eV is carried over from the pure graphite (Figure 4a) into the PVDF graphite combination (Figure 4c). The magnitude of C–OH peaks was reduced in the combined material, and the C–O increased, when compared to the pure PVDF sample (Figure 4b) [19]. The somewhat unexpected part here is the presence of peaks in graphite at around 527 eV, and 535 eV in pure PVDF. These are rather uncommon, and after an extensive search they have been identified as absorbed oxygen [20] and adsorbed water [21], respectively. Their presence is virtually eliminated in the combined material, supporting this opinion.

**Figure 5:**
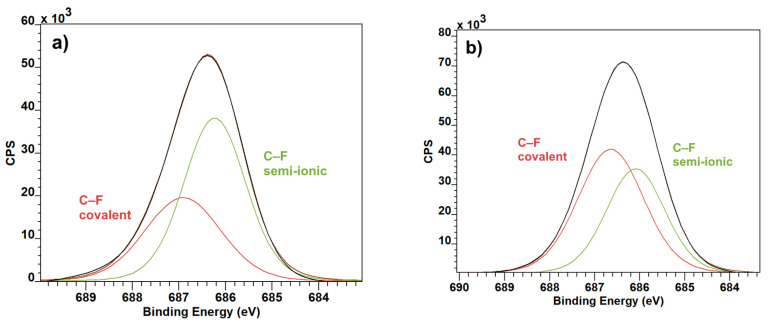
F1 XPS spectra: PVDF (**a**) and PVDF with Graphite (**b**).

The F1 spectra show a change in the dynamic of covalent and semi-ionic bonds after the introduction of graphite. In pure PVDF (Figure 5a), the more prevalent of these two are the semi-ionic bonds, whereas in the combined material, the ratio shifts in the favor of covalent bonds (Figure 5b) [22].

**Figure 6:**
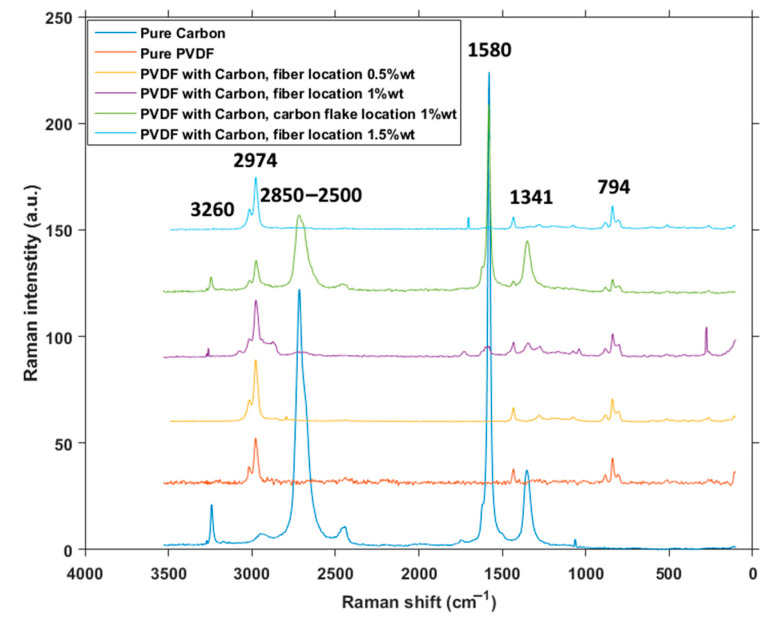
Raman wide spectra.

Figure 6 shows the wide Raman spectra taken from pure carbon, pure PVDF, and combined material from locations of a fiber and of a flake. D-band at 1341 cm^−1^ is activated by the presence of disorder in graphite and is clearly visible in pure carbon and combined material taken at the place of a graphite flake. The most prominent peak in the spectrum is located at 1580 cm^−1^ in carbon and at graphite flake location of PVDF with carbon and belongs to a G band representing in-plane vibrations of C–C bond [23]. Smaller peaks from 2500 to 2850 belong to the 2D group [24], and the last peak belonging to carbon at 3260 cm^−1^ belongs to a G + D’-combined band [25]. Band located around 794 cm^−1^ is attributed to CH_2_ rocking, associated with both α and β phases of PVDF, whereas the band at around 2974 cm^−1^ is assigned to CH_2_ symmetric stretching, associated with β phase [26]. Both these bands are, as expected, present only in material containing PVDF.

**Figure 7:**
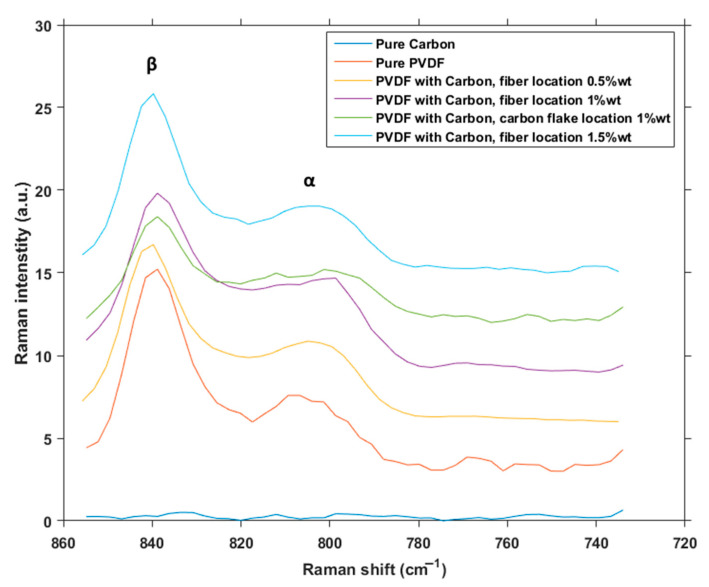
Raman spectra focused on locations of phases.

In the focused Raman spectrum (Figure 7) there are peaks commonly associated with vibration of CH_2_ and CF_2_, corresponding with α and β phases of PVDF [27]. The signal gained from pure carbon in this spectral range is virtually non-existent, which in itself is advantageous for this purpose, since it does not add any interference to the information about phases of PVDF. When the locations of phase peaks are compared to one another, the α phase peak in pure PVDF material is much more pronounced than in PVDF with carbon flakes. In the combined material, the ratio of α and β phases appears to change even more in the favor of β phase in comparison to pure PVDF. This would mean that the incorporation of carbon flakes into PVDF fibers created by electrospinning increases the concentration of β phase above the initial amount granted by the method itself.

**Figure 8:**
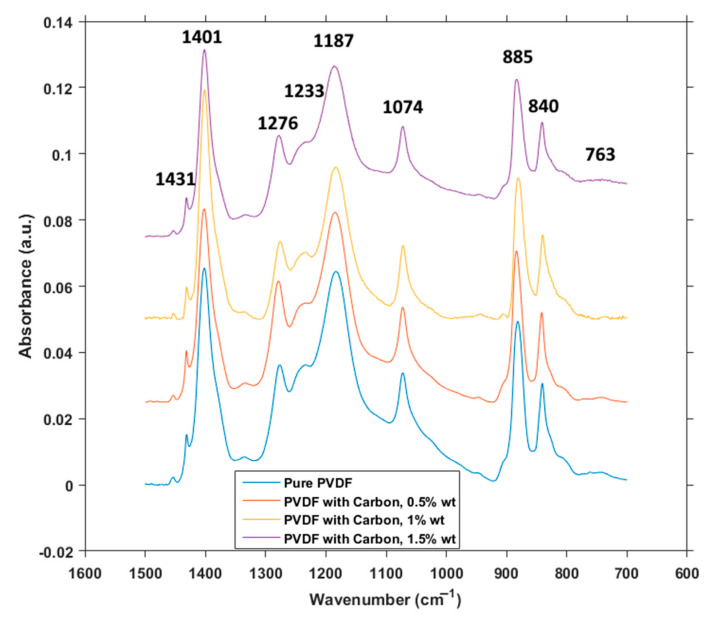
FTIR spectra.

Samples of pure PVDF and PVDF with graphite flakes were subjected to FTIR measurement as well. Reason for this was mainly to compare the concentration of different phases. In Figure 8, it can be seen that, at the first glance, the absorption spectra are almost identical. Locations of virtually all of the peaks remain the same, and their sizes are comparable as well, with a couple of exceptions.

There is a very small and flat peak at 763 cm^−1^, assigned to α phase, but the differences in size correspond with the concentration of this phase in all of the samples. Peak at 840 cm^−1^ is significantly larger as it represents β phase, as do the peaks at 1431 cm^−1^ and 1276 cm^−1^, being characteristic for this crystalline phase. Peak at 1074 cm^−1^ represents mostly the β phase, but references to other phases can be found around this location as well, making the analysis more complicated. From the characteristic peaks there is also 1233 cm^−1^, exclusive for the γ phase. 1187 cm^−1^ is assigned to the combination of β and γ, and the large peaks at 885 cm^−1^ and 1401 cm^−1^ represent the combination of all three phases [28]. Numerical values of concentrations of crystalline phases for each measured content of carbon powder can be seen in Table 1.

The large similarity between presence and locations of the peaks would mean that, since the graphite flakes are not simply scattered on the top of the fibers, but are integrated inside of the material, as was show in Figure 1e,f, the presence of the flakes influences the ratio of crystalline phases on the level of chemical structures. A small peak of α phase can be seen in Figure 8 for pure PVDF, but disappears almost completely in PVDF with carbon flakes, which corresponds with the relative ratio calculation from the absorbance measurements. The increase of γ phase, that is present in the calculations, is however overshadowed by the peak of β phase in Figure 8, as their absorbances share a very close proximity [29]. An effect on the concentration of β phase has been observed by other authors [30] with other materials, like cellulose and carbon nanotubes. In the case of graphite flakes, the large surface area of the graphite acts as a site for heterogeneous nucleation of PVDF during the electrospinning process, which allows the β phase, a crystalline phase more demanding on the conditions of electrospinning process and more prone to destabilization during formation, to crystallize without the increased stress on material caused by uneven and small surfaces [31].

**Figure 9:**
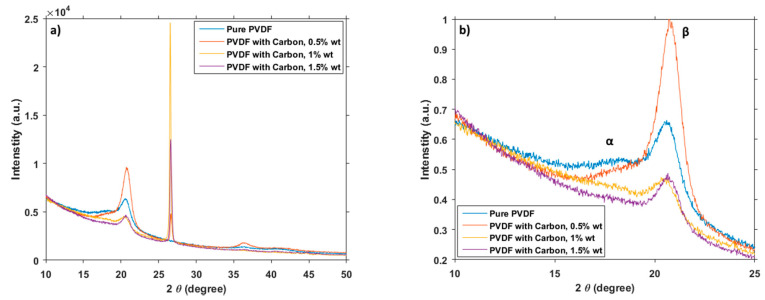
XRD wide spectra (**a**) and focused (**b**).

XRD measurement was performed on samples of pure PVDF and PVDF with graphite flakes. The very first clearly visible thing from the resulting spectra (Figure 9a) is the dominance of carbon peak at around 27°, which overshadows every other peak by far. This is, however, to be expected, as the peak is present only in the material with the carbon flakes and as such serves more as a check for the presence of graphite. More interesting peaks are to be found at around 18° and 21° (Figure 9b), as they belong to α phase and β phase respectively [28]. As with the other measurement methods mentioned here, this result also supports the fact that the ratio of crystalline phases can change by a large amount the PVDF material enriched with carbon flakes in comparison to the pure PVDF.

Dielectric properties of the samples were also measured to get a better idea about the overall behavior of electrical properties. In the case of PVDF polymer, dielectric properties are influenced by the crystalline phase of the material, its orientation and temperature [32,33,34]. Table 1 shows the relation between crystalline phases and measured relative permittivity at 1 kHz. The standard deviation of all the phase concentrations is 5%, corresponding with the claim from the manufacturer of the electrospinning device. From the numbers acquired it is clear, that the dependency between these values is not simple. Because the permittivity was measured under constant temperature, we can disregard its influence on the results in Table 1. We are, then, left with values that, at the first glance, do not have any correlation to one another. Relative permittivity is not directly dependent solely on one of the phases, but rather on all three combined. The greatest upset in the expected values is presented in the material with 1.0 wt% carbon powder content, and its abnormally high concentration of γ phase. This is one of the main influences on the large value of relative permittivity, though not the only one. All three crystalline phases together influence the resulting measured permittivity values in some manner. In addition to the crystallinity, there were also other, better documented influences on the dielectric constant, working against the influence of graphite flakes in the material, such as presence of defects or irregularities in the sample, which tend to cause a higher mobility of dipole moments and the growth of dielectric constant [35].

During the piezoelectricity measurements, however, we have found out, that, while the concentrations of the phases did indeed change, and while concentrations of phases have a well-documented effect on piezoelectric capabilities of PVDF film, we were unable to measure any reasonable piezoelectric response. This is likely because the fibers are not strictly oriented, and any possible piezoelectric effect it can have will cancel itself out with fibers going in all directions, even though in PVDF films the crystalline phases are directly linked to its piezoelectric behavior [36]. In addition, within the fiber, the molecular chains are not strictly oriented either, enhancing this problem further. For this reason, we have chosen to perform triboelectric measurements instead, as they were capable of yielding possible useful data.

**Figure 10:**
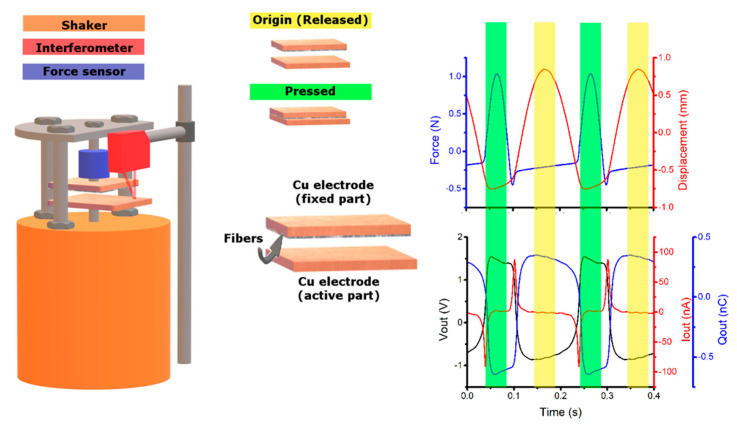
Triboelectric measurement setup. Time dependent chart on the right side shows Force and Displacement during measurement (upper chart—input conditions for the triboelectric nanogenerator) and open circuit voltage Vout, short circuit Iout and charge generated by the sample Qout (lower chart—output electrical performance of the triboelectric nanogenerator).

Triboelectric generator was assembled in vertical contact-separation mode as was mentioned above in section materials and methods. Fixed electrode was made up of a PVDF sample clamped on the side. Active bottom electrode was controlled by a shaker to facilitate the pressure changes in the measurement. Both electrodes were electrically connected by resistor representing load resistance. AC voltage was measured on the load resistance and corresponding DC value was established by root mean square (RMS), as is done by many authors [37,38,39,40]. Output RMS voltage of the triboelectric generator was measured for different values of load. We have decided to measure the power from RMS voltage, without the use of any type of rectification from AC to DC. Then, the output power is not influenced by the type of rectification and the type of diodes, and raw power from material is captured. Maximal average power was evaluated for optimal value of load connected to triboelectric nanogenerator. It is necessary to note that the output power of the generator is strongly influenced by mechanical frequency of operation and mechanical force of the touch of electrodes. We used constant operation conditions for our triboelectric generator, only the triboelectric fiber material inside was changed. In this way, it was possible to compare the triboelectric output capability of the materials and compare them. We used constant frequency of operation of 5 Hz, constant force of the touch of electrodes of 1 N and constant maximal gap between electrodes was 1.6 mm. Measurement setup and record of measurement in time can be seen in Figure 10. Force and Displacement represent input conditions for testing of the materials (upper chart in Figure 10). Force is measured in positive and negative direction. Force in negative direction appears when the upper (fixed) electrode is released, and then the own weight of the electrode is measured by the force meter. Red color shows position of the bottom electrode (active electrode). In Figure 10, we can see that the highest force F = 1 N appears when the electrodes are maximally pressed. Motion of the active electrode is blocked at this position as can be seen from the red line in the Figure 10 (upper chart). The lower chart in the Figure 10 shows electric behavior of the tested sample. The open circuit voltage, short circuit, and charge generated by the sample during operation can be seen in Figure 10.

**Figure 11:**
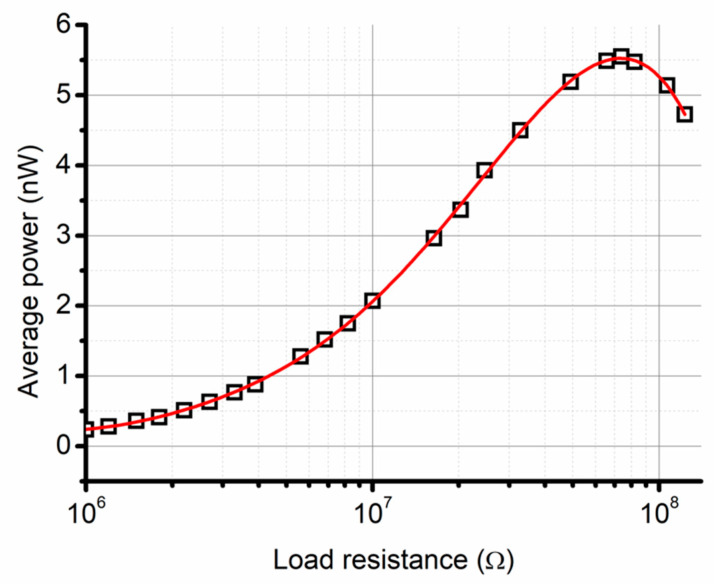
Average power as a function of load connected to the sample PVDF with 0.5% wt carbon powder.

Average power for different values of loads connected to the sample PVDF with 0.5% wt carbon powder is shown in Figure 11, for input conditions of 1 N pressing force, displacement between electrodes at 1.6 mm and frequency of 5 Hz. Optimal load is 70 MΩ for this sample and maximal power is 5.5 nW. This measurement was performed for comparison of our materials between each other from triboelectric point of view, how the content of carbon influences this property. We used low frequency of vibration 5 Hz and low force of touch of the electrodes with consideration to stiffness of the construction on the shaker.

From the measured values (Table 2), it is visible that the average power decreases with the increased concentration of carbon powder. This seems to be a fairly straightforward dependency, be it because of the increase of concentration of foreign material to PVDF in the form of carbon powder, or because of the decrease of β phase concentration with the increased percentage of carbon powder.

## 4. Conclusions

Polyvinylidene fluoride fibers enriched by incorporating carbon flakes inside of them during their weaving display a number of properties differing from pure PVDF material. Results obtained from an array of measurements are greatly affected by the fact that it is not a carbon film coating the fibers, but rather flakes inside of the fiber structure, as demonstrated by SEM. One of the most prominent changes, captured by Raman spectroscopy, FTIR, and XRD, is the change in crystalline phase concentration. Incorporating carbon flakes appears modify the concentrations of α, β and γ phases greatly. Carbon itself does not add any peaks in Raman spectroscopy and FTIR, and in XRD it is visible at one location only, even if the peak is quite big in comparison to others. XPS measurement managed to detect two important things. The first is the shift of ratio of semi-ionic bonds and covalent bonds, where, in pure PVDF material, semi-ionic bonds are more prevalent, but in the combined material, the ratio shifts in the favor of covalent bonds. This is likely due to fluorine atom bonding to a graphite structure defect, forming a covalent C–F bond. The second is the disappearance of adsorbed oxygen and water from the pure forms of carbon and PVDF, respectively. The reason for this is likely connected with the reforming of bonds in the materials when combined, losing the ability to adsorb water and oxygen in any measurable degree. The introduction of carbon had also an effect on the electric properties of PVDF, drastically changing the dielectric constant, and with an increased content of carbon powder lowering triboelectric properties. The results gathered and described open a new window of opportunity for utilizing polyvinylidene fluoride modified by a cheap and accessible material.

## Figures and Tables

**Table 1:** Percentage concentration of phases and measured relative permittivity at 1 kHz of pure PVDF and varying content of carbon powder.

	Pure PVDF	PVDF with 0.5% wt Carbon Powder	PVDF with 1.0% wt Carbon Powder	PVDF with 1.5% wt Carbon Powder
α phase concentration [%]	13.454	4.3404	4.622	38.287
β phase concentration [%]	82.521	94.631	85.840	60.526
γ phase concentration [%]	4.025	1.029	9.538	1.187
Relative permittivity ε_r_	3.986	1.970	7.998	4.714
Standard deviation of ε_r_	0.201	0.087	1.028	0.935

**Table 2:** Relative permittivity and maximum RMS power from triboelectric measurements of the pure PVDF fibers and those with carbon powder of different concentrations.

	Average Power [nW]	Standard Deviation of the Average Power [nW]
PVDF with 0.5% wt carbon powder	5.5	0.02
PVDF with 1.0% wt carbon powder	4.8	0.04
PVDF with 1.5% wt carbon powder	4.2	0.03
